# Autoregulation in Resistance Training for Lower Limb Tendinopathy: A Potential Method for Addressing Individual Factors, Intervention Issues, and Inadequate Outcomes

**DOI:** 10.3389/fphys.2021.704306

**Published:** 2021-08-05

**Authors:** Ian Burton

**Affiliations:** National Health Service (NHS) Grampian, Aberdeen, United Kingdom

**Keywords:** tendinopathy, resistance training, autoregulation, exercise, musculoskeletal-disorders

## Abstract

Musculoskeletal disorders, such as tendinopathy, are placing an increasing burden on society and health systems. Tendinopathy accounts for up to 30% of musculoskeletal disorders, with a high incidence in athletes and the general population. Although resistance training has shown short-term effectiveness in the treatment of lower limb tendinopathy, more comprehensive exercise protocols and progression methods are required due to poor long-term outcomes. The most common resistance training protocols are predetermined and standardized, which presents significant limitations. Current standardized protocols do not adhere to scientific resistance training principles, consider individual factors, or take the importance of individualized training into account. Resistance training programs in case of tendinopathy are currently not achieving the required intensity and dosage, leading to high recurrence rates. Therefore, better methods for individualizing and progressing resistance training are required to improve outcomes. One potential method is autoregulation, which allows individuals to progress training at their own rate, taking individual factors into account. Despite the finding of their effectiveness in increasing the strength of healthy athletes, autoregulation methods have not been investigated in case of tendinopathy. The purpose of this narrative review was 3-fold: firstly, to give an overview and a critical analysis of the individual factors involved in tendinopathy and current resistance training protocols and their limitations. Secondly, to give an overview of the history, methods, and application of autoregulation strategies both in sports performance and physiotherapy. Finally, a theoretical adaptation of a current tendinopathy resistance training protocol using autoregulation methods is presented, providing an example of how the method could be implemented in clinical practice or future research.

## Key Points

- Specific resistance training protocols for tendinopathy rehabilitation have been widely adopted in clinical practice and research for more than 20 years. However, the protocols remain largely underdeveloped and have evolved little since their initial development. Current protocols lacking adherence to the scientific principles of progressive resistance training perhaps contribute to their poor long-term outcomes. There is an increasing attention being paid to the application of the scientific principles that underlie an adaptation to manipulating the exercise stimulus in a progressive manner, which potentially improve training prescription and outcomes in case of tendinopathy.
- A significant range of physical, genetic, and psychological individual factors has been identified across patients with tendinopathy, resulting in a large heterogenous population. Tendinopathies are also present in various stages of a continuum, with each stage potentially responding differently to the training stimulus. These factors can impact on individual performance, adaptation, adherence, and response to resistance training interventions. Therefore, protocols should be personalized and individually modified as opposed to the current predetermined and standardized protocols.
- Autoregulation represents a potential method of personalizing tendinopathy resistance training protocols, allowing for a modification based on the individual training response across the tendinopathy continuum. Future research should examine the efficacy of such an approach, like the example presented here, as a potential method of improving the outcomes of common tendinopathy resistance training interventions.

## Introduction

Recently published studies have highlighted how musculoskeletal disorders are collectively a leading global cause of disability and pain, impacting on a broad demographic spectrum of society (Vavken and Dorotka, 2011; Moraes et al., 2014; Smith et al., 2014; Hoy et al., 2015; Onuora, 2019). Tendinopathy accounts for up to 50% of all musculoskeletal disorders and is often associated with chronic pain, decreased physical function, and reduced participation in activities (Maffulli et al., 2003; Dos Santos Franco et al., 2019). Although the etiology of tendinopathy is still not fully understood, it is known to be caused by repetitive tendon microtrauma and failed tendon healing (Magnussen et al., 2009; Fu et al., 2010; Lui et al., 2011; Simpson et al., 2016; Magnusson and Kjaer, 2019). This disrupted healing process manifests as increased tendon thickness, neovascularization, and the incidence of calcium deposits or calcification (Andres and Murrell, 2008; Rui et al., 2011). The previously used term “tendinitis” is referred to an inflammatory process, and, in case of the presence of inflammatory cells, the term tendinopathy has been adopted to reflect the often degenerative and non-inflammatory nature of the disorder (Jarvinen et al., 2005; Sharma and Maffulli, 2005; Rees et al., 2014). Athletes exposed to frequent loading through training and competition are particularly susceptible to developing tendinopathy due to repeated tendon stress and overloading (Ganestam et al., 2016). Patellar tendinopathy is reported to have a prevalence of 45% in elite volleyball players and 32% in elite basketball players (Abat et al., 2017). The lifetime risk of developing Achilles tendinopathy in elite runners amounts to 52%, reporting an incidence of 24% among competitive athletes, and 18% among young athletes compared to that in sedentary populations amounting to 5.9% (Ahmad et al., 2013).

A diverse range of common treatments, such as anti-inflammatory medications, corticosteroid injections, low-level laser therapy, ultrasound, platelet-rich plasma injections, prolotherapy, glycerol trinitrate patches, manual therapy, shockwave therapy, and exercise, are used in tendinopathy rehabilitation (Cardoso et al., 2019; Girgis and Duarte, 2020). Exercise is the most common and recommended treatment option, including heavy slow resistance training (HSRT) and eccentric strengthening interventions having positive outcomes for various lower limb tendinopathies such as plantar heel pain (PHP), Achilles, and patellar tendinopathy (Dimitrios, 2015; Riel et al., 2019b). Despite positive short-term outcomes, long-term outcomes remain poor, with up to 40% of patients having symptoms 2 years after resistance training interventions (Rathleff and Thorborg, 2015). Recent evidence suggests that current resistance training interventions have not achieved the required loading dosages to stimulate mechano-biological changes, such as increases in strength and changes in tendon (Riel et al., 2019a). Current resistance training interventions may not follow scientific training principles and may require more accurate modification of training variables, such as frequency, intensity, and volume (Abat et al., 2017). A shift to a more scientifically supported training model, such as periodization models used in training athletes, may improve outcomes (Fleck, 2011). Autoregulation is a concept of training derived from periodization models, which are related to manipulating the training variables based on the circumstances of an individual, such as response to training and daily readiness to train (Mann et al., 2010).

Recently, several individual factors have been identified in case of tendinopathy, highlighting the heterogeneity of this patient population (Steinmann et al., 2020). Therefore, it is likely not appropriate that all patients receive the same rigid training protocol in the standardized resistance training. The implementation of autoregulation methods during tendinopathy resistance training may achieve the individualization of training loads and dosage, which may maximize clinical outcomes compared to the following standardized protocols not being tailored to the individual (Helms et al., 2018). However, to the authors' knowledge, autoregulation methods during tendinopathy resistance training interventions have not previously been investigated. Therefore, the purpose of this review is to synthesize the current research on resistance training interventions for lower limb tendinopathies, their limitations, and potential autoregulation strategies. The review begins with an overview of individual factors and resistance training protocols in tendinopathy before providing an overview of the theory and implementation of autoregulation methods. Finally, an overview of the potential application of autoregulation methods will be applied to a current tendinopathy protocol, allowing for a potential clinical application to improve clinical outcomes.

## Individual Factors

Tendinopathies are challenging to treat and are considered to have a multifactorial pathogenesis resulting from extrinsic and intrinsic factors (Seitz et al., 2011; Deans et al., 2012). Abnormal or repeated tendon loading factors combined with genetic, biological, and cellular variations may lead to tendon changes and damage (Wu et al., 2017). Despite a condition that is considered to be common in athletes and physically active, tendinopathies are also commonly seen in sedentary patients and the elderly (Moosmayer et al., 2009). Gender differences may also present in case of Achilles tendon injuries, that is, being more common in men than women (Andrew and Jonathan, 2014). Tendinopathies in different locations, i.e., upper and lower limbs, present different pathophysiological and etiological circumstances, which will require individual treatment approaches despite the treatment often being standardized across all tendinopathies (Clayton and Court-Brown, 2008).

Individual variability in biomechanics, such as anatomical differences, malalignment, neuromuscular capacity, weakness or strength, foot structure, flexibility, repeated training errors, and movement kinetics and kinematics may lead to repetitive, excessive, or compressive loading on tendons (Benjamin et al., 2008; Sharma and Maffulli, 2008; Dean et al., 2017; Ferreira et al., 2020). In patellar tendinopathy, almost 37 different individual biomechanical risk variables have been identified in relation to jump-landing strategy alone (Harris et al., 2020). The identified external risk factors may have a complex interplay with individual intrinsic and biological factors, namely age, gender, bodyweight, hormones, genetics, height, prior tendon injury, and health conditions such as obesity, diabetes, hyperlipidemia, rheumatological conditions, and osteoarthritis (Kjaer et al., 2006, 2009; Leblanc et al., 2017). Environmental factors, such as daily activities, smoking, alcohol consumption, poor nutrition or sleep habits, physically demanding workplace, stress, inappropriate footwear, and medications such as corticosteroids, statins, aromatase inhibitors, and fluoroquinolone antibiotics, may also contribute to the individual tendinopathy risk (van der Linden et al., 2002; Knobloch, 2016). Individual differences and factors, such as loading capacity and tolerance, motor control, education, beliefs, exercise types and preferences, and psychosocial and contextual factors, have also been shown to be important factors related to the individual response to rehabilitation and treatment outcomes (Silbernagel et al., 2007a; Kongsgaard et al., 2010; Littlewood et al., 2012; Cook and Docking, 2015; Hanratty et al., 2016; O'Keeffe et al., 2016; Thiese et al., 2016; Dejaco et al., 2017; Gillespie et al., 2017; Ingwersen et al., 2017; Mallows et al., 2017a,b; Mc Auliffe et al., 2017; Raschhofer et al., 2017; Plinsinga et al., 2020).

Andia and Maffulli (2019) highlighted that the response to tendinopathy treatment interventions is strictly individualistic and can be inherent to the genetic background, demography, immune status, microbiota function, associated metabolic diseases, systemic drugs or local anesthetics, the characteristics and stages of recipient tissue, including inflammation, angiogenic state, and cellularity. Positive responses to resistance training are influenced by a multitude of individual factors, including fitness level, training history, nutritional intake, psychological and social states, sleep and recovery, age, weight, and overall training workload (Pickering and Kiely, 2019). However, some of these factors may prove difficult to evaluate in clinical practice (Abate et al., 2020), with a thorough clinical assessment of each individual and the requirement of their personal factors prior to prescribing resistance training interventions (Maestroni et al., 2020). A wide variety of potential factors involved in the development, progression, and rehabilitation of tendinopathy leads to a highly diverse and broad range of potential cellular responses and ultimately a broad pathological spectrum of disease, leading to a vastly heterogenous tendinopathic patient population.

Due to a plethora of potential individual patient characteristics and risk factors involved in any type of tendinopathy, their presentation is highly heterogenous in nature, with a multitude of potential factors contributing to triggering the onset of tendinopathy (Steinmann et al., 2020). Individual patient characteristics (age, gender, and genetics) can interplay with lifestyle habits (smoking, alcohol consumption, and physical activity levels) leading to a diverse spectrum of potential tendinopathy risk and onset factors. All cases of tendinopathy do not clinically present uniformly, with each individual case of tendinopathy having a varying degree of symptomatic presentation of pain, swelling, weakness, and functional ability. Pain experienced from tendinopathy is not always associated with tissue damage, suggesting that other neurophysiological mechanisms may be involved (Rio et al., 2015a, 2016; Hainline et al., 2017). Central nervous system hypersensitivity and the role of pain central sensitization have recently emerged as a potential contributing factor, which can vary in the presentation among individuals (Littlewood et al., 2013a; Plinsinga et al., 2015, 2018; Sanchis et al., 2015).

Although the importance of an individualized approach to a heterogenous tendinopathy population has been repeatedly stressed in the tendinopathy literature, it is clearly a limitation while developing studies due to the effort and the need to homogenize samples. However, there is a special need to make progress in the knowledge of treating patients with tendinopathy even in the case of no possibility of perfect homogeneity (Abat et al., 2017). The heterogeneous presentation of tendinopathies means that a “one-size-fits-all” treatment approach may be inappropriate and related to poor long-term treatment outcomes (Silbernagel, 2014). The Achilles tendon, for example, can experience a positive adaptation when exposed to the strains of a specific range or “sweet spot,” which depends on several factors known to differ between the individuals, requiring treatment interventions that account for these interindividual differences (Pizzolato et al., 2019). Physiotherapists have reported to use complex clinical reasoning to adapt eccentric training research protocols for individual patients. For example, the need to change eccentric loading protocols in patients experiencing pain prevented them from being adherent to the published protocols, and therefore substituting with isotonic or isometric loading initially. To make recommendations clearer, future research should focus on variables of the eccentric training program that could impact on efficacy, such as exercise speed, duration, progression and loading rate, and chronicity and severity of the tendinopathy (Rowe et al., 2012).

As the etiology of tendinopathy is multifactorial, rehabilitation should be focused on identifying the multiple risk factors that affect a specific individual (Peters et al., 2016). It could be considered as a disservice to the patient if the factors contributing to the development of their tendinopathy are not addressed (Kulig et al., 2015). It is important to consider that each individual will respond differently to rehabilitation, and each program must be tailored to the individual with “Recipe” approaches to tendinopathy rehabilitation failing to capitalize on the potential for recovery (Cook, 2011; Cook and Purdam, 2014). The needs of an individual must be addressed in the rehabilitation program, which was highlighted in a recent e-Delphi survey to establish a patellar tendinopathy rehabilitation framework. The results quantified three central aspects, including functional abilities, individualized rehabilitation, and load tolerance, which formed the foundation of the rehabilitation framework (Morgan et al., 2018a). Factors relevant, when accessing, adapting, and applying training protocols, to the patient include the mapping information gathered from a subjective assessment to pathophysiological knowledge and likely treatment responses to enable clinical decisions that utilize interventions in an individualized way (Morrissey, 2015). Comorbidities, symptom development, patient beliefs, psychosocial and cultural factors, possible tissue demands, and previous intervention outcomes may influence treatment outcomes. Different tendinopathies and pathological tendon states combined with individual circumstances require an individualized treatment approach, which must be applied to progressive resistance training interventions (Morrissey, 2015).

Despite the necessity of addressing individual factors in tendinopathy, they are often not considered in rehabilitation, with the treatment offered to a young elite male athlete often the same as that for a postmenopausal woman (Cook and Purdam, 2009). Cook and Purdam (2009) proposed that tendinopathy pathology is not absolute and presents on a continuum with three distinct stages, with each having implications for the treatment. The staging model acknowledges the heterogeneity of tendinopathy presentation, with the three phases being (1) early reactive tendinopathy due to acute overload, (2) tendon disrepair after failed healing responses, and (3) degenerative tendinopathy with permanent structural and cellular damage (Cook et al., 2016). The individual tendinopathy risk factors discussed earlier may play a role in altering the progression throughout this continuum and will possibly contribute to individual treatment response (September et al., 2008). The tendinopathy continuum model recommends that rehabilitation is optimized by individually tailored treatment to the tendinopathy stage and targeting the primary driver and interrelated alterations. While exercise is considered essential in tendinopathy rehabilitation, a plethora of other treatment options for tendinopathy exists, which can increase the complexity of the clinical decision-making process. Tendinopathies have a heterogeneous clinical presentation due to the variable changes that exist between the individuals in a cellular matrix structure, pain, function, and disability (Malliaras et al., 2010; Malliaras and Cook, 2011). Phenotyping of patients based on multifactorial components of pain, disability, and load capacity and tolerance, may allow clinicians to direct appropriate interventions at such critical limiting factors (Cook et al., 2016). A recent model proposed by Steinmann et al. (2020) has demonstrated the complex interplay of individual factors in tendinopathy pathogenesis and how they relate to the tendon continuum model. A further step would be the integration of individualized treatment strategies into this model in each pathology stage.

## Resistance Training in Tendinopathy

### Resistance Training Protocols

Stanish et al. (1986) were the first to publish on the concept of a predominately eccentric-based resistance training protocol for the treatment of lower limb tendinopathy. However, the publication of the Alfredson eccentric heel-drop protocol for Achilles tendinopathy in 1998 had helped to popularize the concept of using an eccentric-based training protocol for treating Achilles tendinopathy (Alfredson et al., 1998). Following the publication of the Alfredson protocol, eccentric-focused resistance training has become a dominant exercise-based strategy for lower limb tendinopathies for the last 20 years (Malliaras et al., 2013). There are, however, some important differences between these protocols, with Stanish recommending that patients perform the eccentric exercises with no pain, and, on the other hand, Alfredson recommended pushing through pain (Sayana and Maffulli, 2007). The parameters of the Alfredson protocol have also been adapted in several studies in the form of an eccentric decline squat protocol to treat patellar tendinopathy (Young et al., 2005; Visnes and Bahr, 2007). Although eccentric training has positive effects in treating tendinopathies, there are several issues related to eccentric-based training protocols. Despite widespread clinical adoption, there is a dearth of high-quality research confirming that removal of the concentric action from isotonic contractions is appropriate or more useful than full isotonic contractions when treating chronic tendinopathies (Couppe et al., 2015). However, for Achilles (Mafi et al., 2001), patellar (Jonsson and Alfredson, 2005), and tennis elbow tendinopathies (Peterson et al., 2014), eccentric-only training has shown better results when directly compared with concentric-only.

Despite the results from individual studies, comprehensive systematic reviews have consistently concluded that high quality pooling evidence of positive clinical outcomes for isolated eccentric resistance training in lower limb tendinopathies is lacking (Lim and Wong, 2018; Murphy et al., 2019; Mendonca et al., 2020b). The original study on the Alfredson eccentric protocol was conducted with a cohort of athletes, which may be related to the high success rate of the protocol. However, these outcomes may not be generalizable to the general population or sedentary demographics, and it is clear from previous studies that different patient characteristics (activity level and age) potentially have an impact on the response and outcomes from the eccentric-based training (Sayana and Maffulli, 2007). For example, Sayana and Maffulli (2007) found that 45% of the patients in a general population had a failed treatment outcome with eccentric training based on pain and function outcome measures, which suggests that eccentric-only training may not be suitable for all types of patients. However, other single and combined contraction types of resistance training have been investigated, namely concentric-eccentric, isolated concentric, and combined plyometric training (Silbernagel et al., 2020).

Heavy slow resistance training with heavy-load isotonic contractions is a more recent conception in tendinopathy rehabilitation, which has been widely adopted in both clinical research and practice (Malliaras et al., 2013). The HSRT approach was first investigated in case of patellar tendinopathy and was found to have significant positive outcomes for pain and function improvement (Kongsgaard et al., 2009). Similar HSRT interventions based on the same loading and progression parameters were recently investigated with the positive outcomes in Achilles tendinopathy (Beyer et al., 2015) and PHP (Rathleff et al., 2015a). The Alfredson eccentric protocol involves a strict fixed-loading protocol of 180 repetitions and 2 training sessions daily. However, the HSRT protocols can be considered to adhere more to scientific resistance training principles, including progressive increases in volume and load, with increased rest periods between the sessions (Table 1). Individual RCTs have found that HSRT protocols have superior clinical outcomes for pain and function and greater patient satisfactory outcomes compared to eccentric-only training for the treatment of lower limb tendinopathies (Kongsgaard et al., 2009; Beyer et al., 2015; Rathleff et al., 2015a). Despite of possessing positive outcomes, it has been suggested that even HSRT protocols do not achieve the intended dosage levels for tendon adaptation and may be enhanced by improved loading and progression methods, including adopting an individualized intervention rather than a standardized protocol (Riel et al., 2019a). Slow performing isotonic contractions during HSRT interventions are thought to stimulate tendon adaptations by providing high loads and forces across tendons, which may improve tendon compliance and activate remodeling processes when applied progressively, including reducing neovascularization and increasing collagen production (Murtaugh and Ihm, 2013). It is well-known that exercise can influence human cell homeostasis and activate tissue changes and healing in both muscles and tendons, through the process known as mechano-transduction (Khan and Scott, 2009). Despite this capability of exercise and particularly HSRT, it has been suggested that the symptom improvement seen in HSRT protocols may not be due to the changes in tendon architecture but rather improved mechanical properties of the tendon (Farnqvist et al., 2020). Resistance training has also been shown to be more effective than stretching for improving flexibility, reducing the injury risk inducing length changes in short muscles *via* sarcomerogenesis (Weppler and Magnusson, 2010; O'Sullivan et al., 2012; Askling et al., 2013; Konrad and Tilp, 2014; Freitas and Mil-Homens, 2015; Timmins et al., 2016).

**Table 1:** Evidence-based resistance training protocols in lower limb tendinopathy.

**Protocol**	**Tendinopathy**	**Exercise type**	**Sets, repetitions**	**Frequency**	**Duration**	**Progression**	**Pain**
**Characteristics of resistance training protocols in lower limb tendinopathy**							
Stanish et al. (1986)	Achilles	Eccentric-concentric, power	3, 10–20	Daily	12 weeks	Speed then load	Enough load to be painful in 3rd set
Alfredson et al. (1998)	Achilles	Eccentric	3, 15	2 × daily	12 weeks	Increase load as able (backpack)	Enough load to achieve moderate pain
Silbernagel et al. (2007b)	Achilles	Eccentric-concentric, balance, plyometric	Various	Daily	12 weeks	Volume, type of exercise	Acceptable within defined limits
Beyer et al. (2015)	Achilles	Eccentric-concentric (HSRT)	3–4, 15–6	3 × week	12 weeks	15–6RM, increase load as able (external weight machine)	Acceptable if not worse after exercise
Rathleff et al. (2015a)	Plantar heel	Eccentric-concentric (HSRT)	3–5, 12–8	3 × week	12 weeks	12–8RM, Increase load as able (backpack)	Acceptable if not worse after exercise
Kongsgaard et al. (2009)	Patellar	Eccentric-concentric (HSRT)	4, 15–6	3 × week	12 weeks	15–6RM, Increase load as able (external weight machine)	Acceptable if not worse after exercise

*RM, repetition maximum; HSRT, Heavy slow resistance training*.

### Loading Issues

There are more questions still to be answered concerning the appropriate dosage of exercise for tendinopathy, which requires further investigation on the optimal dosage and methods to better individualize resistance training interventions (Silbernagel, 2014). It appears to be a tendency during the lower limb tendinopathy rehabilitation to dismiss exercise as a sufficient treatment option if patients do not respond to the one-size-fits-all exercise program and instead pursue other more costly and invasive treatment options (Silbernagel, 2014). To suitably address individual needs, resistance training prescription and dosage need to be individualized to ensure that it is adequate and appropriate. The staging of tendinopathy and subsequent implications on exercise selection are the other areas that require the consideration of an individual (Barratt et al., 2017). At present, there is insufficient evidence to define the exact speed, intensity, and duration of resistance training needed or to predict, certainly, the long-term effects of resistance training for lower limb tendinopathy. It is relatively common for patients with Achilles tendinopathy to fail to respond to the conservative treatment. For example, in an 8-year study of 83 patients with chronic Achilles tendinopathy, 29% failed conservative management and required surgical intervention (Paavola et al., 2000). The individualization of training progression is also required with the selection of appropriate loads for the individual patient required to attenuate for their sport and performance level. Individual progression of resistance training to plyometric exercises is needed to effectively progress athletes to return-to-sport training volume and intensity (Malliaras et al., 2015).

The overload principle states that it is necessary to stress biological tissues beyond their current thresholds to increase their tolerance to subsequent stresses and avoid future injuries. Training with heavier loads may produce greater load tolerance of tendons, contributing to the positive effects of tissue remodeling and improved structural properties of the degenerated tendons (Kulig et al., 2009). However, it is unlikely that the sufficiently high training loads required for these adaptations are being achieved with the Alfredson protocol due to the parameter of two times daily training with no rest days for 12 weeks. It is clear from the literature on tendon responses to loading that no more than three high-intensity resistance training sessions should be undertaken within 1 week in tendinopathy, to allow adequate collagen synthesis, remodeling, adaptation, and tendon recovery (Malliaras et al., 2015; Magnusson and Kjaer, 2019). It is essential that healing tendons receive adequate rest and recovery time during rehabilitation to allow the anabolic and catabolic processes involved in tendon remodeling and adaptation to occur successfully (Waugh et al., 2018). Sufficient tendon adaptations and improved clinical outcomes from HSRT protocols may also require longer than the current gold-standard intervention time of 12 weeks in lower limb tendinopathy rehabilitation (Sussmilch-Leitch et al., 2012). It is believed that the exercise load has to be relatively high to stimulate tendon remodeling, which can explain why forced eccentric toe-raises, giving high loads, also give better results than using regular concentric/eccentric toe-raises. There is, however, no direct research showing that high eccentric loading is the best option for lower limb tendinopathies (Silbernagel et al., 2001). The dosage parameters (e.g., training frequency, the number of repetitions and sets, rest, and the duration of a contraction) and the types of exercise are the important characteristics of an exercise program, with loads as high as 80% of 1 repetition maximum (RM) being used for isometric (80% 1RM) and 80% of 8RM for isotonic exercises in some studies (van Ark et al., 2016). Tendons require high load per repetition to derive more optimal tendon healing effects with a high percentage of RM in leg extension exercises, which are also shown to improve neural activation and increase muscle strength (van Ark et al., 2016). However, the optimal dosage and loading strategy for positive outcomes, along with a precise mechanism of the effect of heavy loading, remain to be elucidated and require further investigation (van Ark et al., 2016).

Several studies have advocated the need for higher tendon loading in lower limb tendinopathy rehabilitation programs with the type of load even being considered inconsequential (Cuddeford et al., 2018). In most studies, the Alfredson protocol is the most commonly used protocol and consists of up to 180 repetitions daily, with the addition of additional weight *via* a backpack. However, performing an eccentric exercise with a backpack in excess of 20 kg requires significant therapist involvement and may be one of the reasons of only using bodyweight with high repetitions in most studies (Cuddeford et al., 2018). Adequate adherence to training programs may also be falsely reported in studies as it has been found that accurate recording of dosage in participant training diaries is often not performed or misinterpreted. The assumption that active individuals or athletes have a better compliance for the prescribed exercises than non-active individuals, which may also play a role as the studies involving athletes have previously had better outcomes from resistance training interventions in tendinopathy (Alfredson et al., 1998; Sayana and Maffulli, 2007; de Jonge et al., 2010). Despite this, in one study conducted with an athletic cohort, over one-quarter of patients reported performing the exercises at an intensity that was less than half of what was initially prescribed (Abate et al., 2020). Regardless of the intervention population, individualized exercise programs should be adequately supervised, reviewed, and progressed to ensure the adherence and resolution of the tendinopathy (Vicenzino, 2015). The effectiveness of resistance training interventions may be enhanced by implementing intensive care taking procedures, such as having patients to perform exercises with supervision four times per week for 8 weeks as opposed to at home and unsupervised (Stergioulas et al., 2008).

There is a lack of consensus in the literature regarding which training variables may influence the training outcomes, such as whether exercise should be painful, exercise speed and intensity, training duration, progression methods, and unsupervised home vs. supervised clinic-based training (Rompe et al., 2009). Large randomized controlled trials with blinded assessors and long-term follow-up periods investigating differences in these training parameters are warranted. Resistance training at high-intensity levels alongside gradual increases in loading is considered essential to create positive effects and adaptation in human muscles and tendons. Although the importance of loading in tendinopathy rehabilitation through heavy resistance training is well-established, the optimal intervention parameters and dosages require further research investigation and may include the consideration of motor control, biomechanics, kinetic chain, and reactive strength (Silbernagel et al., 2007b). The outcomes of different resistance training approaches in tendinopathy need to be investigated before accurate clinical practice recommendations can be made, with the outcomes for one specific tendinopathy likely not being generalizable to other tendinopathies (Riel et al., 2018). As a previous study on lower limb tendinopathies has not found the superiority of one specific resistance training approach, the long-term effects of different resistance training protocols in tendinopathy remain to be investigated (Riel et al., 2018). Current interventions may also not be prescribing true resistance training, with low-load eccentric protocols perhaps being better described as active stretching due to their high frequency and low intensity (Purdam et al., 2004; Abat et al., 2017). It appears from the literature that only HSRT can be identified as true resistance training as it produces the minimum level of load and tension based on scientific resistance training principles. To cause tendon adaptations, including mechanical, material, and morphological tendon changes, research has suggested that an intensity threshold of >70% of the maximum is required to derive adaptations (Bohm et al., 2015; Arampatzis et al., 2020). High repetitions with inadequate loading in current protocols may be related to their poor long-term outcomes. Alternative strategies of performing more sets with fewer repetitions may allow for sufficient loading, volume, and intensity throughout the training to stimulate adaptations (Davies et al., 2020).

### Kinetic Chain Strength Issues

Strengthening of the entire kinetic chain, particularly, the gluteal muscles in tendinopathy rehabilitation has been advocated considering its successful application to a plethora of musculoskeletal disorders with similar etiologies (Nakagawa et al., 2008; Cibulka et al., 2009; Meira and Brumitt, 2011; Delitto et al., 2012; Khayambashi et al., 2012; Reiman et al., 2012; Barton et al., 2013, 2019; Rathleff et al., 2014; Baldon Rde et al., 2015; Lack et al., 2015; Rio et al., 2015b; Thomson et al., 2016; Mucha et al., 2017). Gluteal strengthening for the loss of lumbopelvic control impairing motor control and the kinetic chain has been recommended in patellar and Achilles tendinopathies (Kountouris and Cook, 2007). Recently, several studies have shown the improvements in pain and function from the targeted hip strengthening exercises in PHP (Kamonseki et al., 2016; Santos et al., 2016; Lee et al., 2019). Hip muscle weakness can cause an excessive adduction and medial rotation of the hip causing dynamic knee valgus and over pronation (Powers, 2003; Khamis and Yizhar, 2007; Barton et al., 2012). Reduction in the strength of the hip abductors and rotators is identified as a risk factor for lower limb tendinopathy due to altered biomechanics (Harty et al., 2005; McPoil et al., 2008; Labovitz et al., 2011; Bolivar et al., 2013; Beeson, 2014; Martin et al., 2014). Therefore, strengthening these muscles could improve dynamic lower limb alignment and could reduce the excessive strain on lower limb tissues (Snyder et al., 2009; Fukuda et al., 2010; Dolak et al., 2011). Hip muscle activation can also impact the force production at an ankle joint impacting gait, which may contribute to the lower limb pain (Komura et al., 2004; Wainner et al., 2007).

Some authors have recommended that isotonic resistance training interventions are superior to eccentric-biased training in lower limb tendinopathy, particularly for those with concentric muscle weakness that would not be addressed with primarily eccentric training. Individual differences and heterogeneity in tendinopathy presentation necessitate assessing and targeting the kinetic chain weaknesses of individuals, which would not be targeted with eccentric-only training (Goom et al., 2016). For example, athletes with patellar tendinopathy have been shown to respond better to comprehensive resistance training interventions, which include addressing the muscle-tendon strength and function of the kinetic chain (Zwerver et al., 2011). To optimally manage tendinopathies, overall functional capacity should be assessed to facilitate progressive overload of the tendon itself and the kinetic chain (Thompson, 2017). Failure to adequately address isolated muscle deficits, kinetic chain deficits or biomechanics may lead to a suboptimal rehabilitation in tendinopathy (Malliaras et al., 2015). Therefore, it could be considered as essential to address any intrinsic or extrinsic risk factor individually during rehabilitation and consider making environmental adaptations if warranted (Morgan et al., 2018b).

Lower limb tendinopathy rehabilitation may be enhanced by the presence of progressive loading of an muscle-tendon unit alongside targeting the whole lower extremity and kinetic chain. However, an optimal loading protocol to improve outcomes is unclear at present (Cook, 2018). For athletes, individual needs can be addressed through a comprehensive evaluation by physiotherapists to identify particular areas of weakness in the biomechanical kinetic chain (Rudavsky and Cook, 2014). Developing an individualized rehabilitation protocol must consider that elite athletes have increased training and competition demands and will require more comprehensive rehabilitation than amateur athletes for a successful return to sport and avoiding a relapse of the tendinopathy (Rudavsky and Cook, 2014). It may be considered as essential to address any whole body strength deficits in athletes with tendinopathy due to their increased physical loading demands (Kulig et al., 2015). Patellar tendinopathy rehabilitation based on individualizing the rehabilitation intervention according to the participant's specific biomechanical deficiencies and load tolerance has demonstrated an improvement in pain and functionality in elite rugby union players with patellar tendinopathy (Morgan et al., 2018a).

Hip extensor muscles such as the gluteus maximus has been considered as an important target for increasing strength and function during lower limb tendinopathy rehabilitation (Scattone Silva et al., 2015). Previous research has found an association with weak hip extensor muscles and poor lumbopelvic control and the risk of developing lower limb tendinopathy, which may be due to an altered load distribution on the lower limb kinetic chain (Stasinopoulos and Malliaras, 2016). A recent Delphi study of clinical practice found that there was a strong predisposition (87%) toward hip strengthening as a component of patellar tendinopathy rehabilitation programs (Morgan et al., 2018a). Similarly, a recent survey of Brazilian physical therapist practice for patellar tendinopathy reported that hip strengthening exercises were secondary only to quadriceps eccentric training in terms of the frequency of prescription (Mendonca et al., 2020a). Rehabilitation interventions focusing on improving the hip extensor muscle strength in patellar tendinopathy have shown positive outcomes. An 8-week treatment protocol of jump-landing modification and hip-specific muscle strengthening improved pain, function, and biomechanics in an athlete with patellar tendinopathy (Scattone Silva et al., 2015). Biomechanical studies have found a variety of risk factors, both local and distal to the knee joint, which may be associated with the development of lower limb tendinopathy (Powers, 2010). Physiotherapists must consider these components when tailoring a comprehensive intervention for treating athletes with patellar tendinopathy. The clinical effectiveness of existing rehabilitation strategies in lower limb tendinopathy management options, including eccentric resistance training, do not have satisfactory long-term clinical outcomes. Evidence suggests that not all patient types respond adequately to eccentric-only loading, highlighting the necessity of future research investigating alternative or additional resistance training strategies. Future research on resistance training interventions in tendinopathy should focus on addressing proximal, local, and distal factors including strengthening of the kinetic chain as opposed to isolated strengthening of the pathological tendon (Scattone Silva et al., 2015).

### Reactive Strength Issues

Athletic activities such as running and jumping involve energy storage and release from tendons, supported by strong muscular contractions, known as the stretch-shortening cycle (SSC) (Taube et al., 2012). Plyometric exercise, which utilizes the SSC, is a common training practice among athletes. It has been shown to improve the maximal rate of torque development, maximal voluntary contraction, jump performance, running economy, and leg stiffness in active people (Spurrs et al., 2003; Kubo et al., 2007; Foure et al., 2010). In athletes, lower limb tendinopathy occurs most commonly in those participating in activities involving the SSC, such as running and jumping (Malliaras et al., 2013). During athletic activities, the Achilles tendon experiences loads 6–12 times of bodyweight. These high loads require a considerable strength and power to repeatedly generate force and enable the storage and release of energy during athletic activity (Cook et al., 2016). Therefore, rehabilitation programs focusing solely on strength *via* the use of various heel-raise programs may lead to suboptimal functional capabilities, persistent strength deficits, inadequate rehabilitation, and ultimately high recurrence rates of tendinopathy (Child et al., 2010; Gajhede-Knudsen et al., 2013).

Lack of consensus in physiotherapy of what constitutes strength may be a factor in the predominance of unidimensional rehabilitation programs. Little consensus exists on the presence of strength deficits in tendinopathy, leading to the implementation of ineffective rehabilitation programs focusing on one type of exercise only in clinical practice (McAuliffe et al., 2019). To clarify this uncertainty, several categories of strength have been suggested. Maximal strength involves a maximal force development through high-load and low-velocity movements. In contrast, explosive strength is considered as an ability to rapidly produce muscle force through medium to high-load and high-velocity movements such as the rate of force development. On the other hand, reactive strength is considered as the ability of the gastric-soleus muscle complex to store and release energy through a sufficient function of the SSC through low-load, high-velocity plyometric exercises such as hopping and jumping (Beattie et al., 2014). Deficits in SSC power and efficiency have been shown in Achilles tendinopathy as measured by leg stiffness during submaximal hopping and a reduction in maximal hopping distance (Maquirriain, 2012; Wang et al., 2012). After 6 months of recommended exercise rehabilitation in Achilles tendinopathy without plyometric exercise, persistent SSC impairments have been found after 1 year (Silbernagel et al., 2007a).

Heavy slow resistance training in healthy individuals has been found to increase tendon stiffness, whereas plyometric training increases joint stiffness and jumping performance (Kubo et al., 2007). This suggests that the plyometric exercise likely has complimentary effects to HSRT in tendinopathy rehabilitation and may reduce persistent SSC deficits, which can decrease rehabilitation effectiveness. A consistent barrier to implementing the plyometric exercise for lower limb tendinopathy in rehabilitation is that it is not implemented progressively and therefore aggravates symptoms (Chmielewski et al., 2006; Kountouris and Cook, 2007). The common belief that plyometric activities will have a negative impact on tendinopathy rehabilitation is not supported by evidence and it may be beneficial for improving outcomes (McAuliffe et al., 2019). In those with Achilles tendinopathy who continued plyometric exercise such as sprinting, jumping, and hopping, similar pain and function were reported to those who stopped all plyometric activities (Silbernagel et al., 2007b). This study identified that SSC loads are tolerable during tendinopathy rehabilitation in active people, provided they are guided by a pain- and symptom-monitoring model (Silbernagel et al., 2007b). Another study in Achilles tendinopathy used a loading intervention integrating plyometric exercise for 12 weeks to address the entire strength spectrum, which resulted in improvements in pain and disability at 12 months (Silbernagel et al., 2007a). Further studies integrating plyometric training in tendinopathy with traditional HSRT are required, particularly in PHP as it has yet to be investigated (Huffer et al., 2017).

A recent systematic review identified that individuals with Achilles tendinopathy demonstrate strength deficits across the strength spectrum of maximal, reactive, and explosive compared to the uninjured side or asymptomatic controls (McAuliffe et al., 2019). This study concluded that the current focus on maximal strength in tendinopathy rehabilitation and the lack of emphasis on reactive or explosive strength may not optimally match the entire strength spectrum. The authors argued that this could explain why Achilles tendinopathy rehabilitation is only moderately effective with recurrence as high as 27% and why SSC deficits persist post rehabilitation (van der Plas et al., 2012). Similarly, the lack of emphasis on plyometric training in PHP rehabilitation may also help to explain why the rehabilitation focused only on strengthening HSRT is only moderately effective (Riel et al., 2019a). Approximately 40% of the patients with PHP have pain and symptoms 2 years after the diagnosis and treatment, suggesting that new and effective treatments are warranted (Rathleff et al., 2015a). Training only one aspect of the strength profile of an individual, which is common in the lower limb tendinopathy rehabilitation with concentric-eccentric HSRT, will likely not optimally improve the performance across the entire strength spectrum (Allison and Purdam, 2009). However, only Silbernagel et al. (2007a) have addressed the entire strength spectrum in Achilles tendinopathy rehabilitation, with no studies currently addressing it with PHP (Huffer et al., 2017). Plyometric loading could potentially be a more potent stimulus for muscles compared to tendon adaptation, thus it may compliment HSRT programs, which more potently stimulate tendon adaptation (Mersmann et al., 2017). Increased tendon stress and strain due to a non-uniform musculotendinous development have been observed recently in adolescent volleyball athletes, a high-risk group for tendinopathy (Mersmann et al., 2019). Previous findings highlight the importance of improving the current understanding of the interaction of physical maturation and loading, indicating that SSC training *via* plyometric exercise may act as a tendinopathy prevention strategy (McAuliffe et al., 2019).

Recently, a pain-guided progressive hopping intervention was found to be safe and feasible while adding to the currently recommended HSRT exercise and education for Achilles tendinopathy in recreational runners (Sancho et al., 2019b). This study also included kinetic chain isotonic strength exercises for the gluteus medius and maximus as recent studies have shown that weakness and delayed onset of the gluteal muscles are common while running in those with lower limb tendinopathy (Sancho et al., 2019a). Despite providing evidence of plyometric exercise possessing complimentary effects to HSRT and improving rehabilitation outcomes, there is a dearth of studies investigating the effects of plyometric exercise in tendinopathy rehabilitation (McAuliffe et al., 2019). This is perhaps due to the perception by patients and clinicians that plyometric exercise is potentially damaging and counterproductive in tendinopathy rehabilitation (Sancho et al., 2019b). However, providing education on the benefits of exercise for pain can reduce self-reported pain following an acute exercise compared to those not receiving pain education (Jones et al., 2017). The following sections provide an overview of the application of autoregulation limited to strength and conditioning (S&C), before finally considering the potential application of autoregulation in physiotherapy and its integration into current HSRT protocols in tendinopathy.

## Periodization in Resistance Training

In the profession of S&C, the use of periodized programming for training is generally accepted as a more effective way of ensuring strength gains for athletes compared to non-periodized programming (Harries et al., 2015). Periodization can be defined as the planned distribution of training to increase the potential for achieving optimal sports performance at a predetermined time point (Conlon et al., 2016; Kiely, 2018). Block periodization has been shown to effectively improve strength and power in athletes and is based on several distinct training mesocycles; hypertrophic, basic strength, and maximal strength (Issurin, 2008, 2010). The mesocycles are performed in an order with an increase in intensity and a decrease in the overall training volume throughout the model (Bartolomei et al., 2014). Maximal strength is considered one of the most important factors in maximizing athletic performance and has been shown to improve the performance in many running sports and reduce injury risk (Stone et al., 2004; Wisloff et al., 2004; Storen et al., 2008). In physiotherapy, strength exercise is one of the most common treatment prescriptions for musculoskeletal disorders such as tendinopathy (Babatunde et al., 2017; Booth et al., 2017). Although there has been research showing the applicability of using structured periodized training in the rehabilitation of athletes, there is a lack of studies on its use on athletes with tendinopathy or the general population in physiotherapy (Lorenz et al., 2010; Lorenz and Reiman, 2011; Reiman and Lorenz, 2011; Hoover et al., 2016; Ladlow et al., 2020).

Periodized strength training typically manipulates the intensity as a percentage of 1RM, which is obtained prior to beginning the training. This approach does not account for daily fluctuations in the training performance due to fatigue and strength levels, which may affect its accuracy and outcome. There can be significant variations in daily strength as high as 20%, resulting in a variable performance when using a fixed method (Hoover et al., 2016). Individual variability in factors, such as genetics, sleep, nutrition, psychological stress, lifestyle factors, total training load, and heart rate variability, may also affect the training performance (Mann et al., 2014; Costa et al., 2019). Fixed periodization also does not account for the individual training response and as such the potential for strength gains may be greater than the potential this model facilitates (Timmons, 2011; Fisher et al., 2017). Personalized exercise prescription and progression may be a more appropriate method, which better accounts for a large interindividual and biological variability that exists in response to training interventions (Mann et al., 2010). Therefore, the use of a method known as autoregulation during training programs would allow individuals to increase strength at their own capability and can allow a constant adjustment of repetitions, which may prevent plateaus in training (Mann et al., 2010).

## Autoregulation

Autoregulation is a form of periodization, which involves manipulating or adjusting the variables of strength training based on an individual's daily or weekly readiness to train (Mann et al., 2010). Due to the heterogeneous nature and the multifactorial complexities involved in individual responses to training, the implementation of autoregulated training derives improved training adaptations and outcomes compared to traditional fixed training models (Zourdos et al., 2016a; Rauch et al., 2020). Although autoregulation originated in physiotherapy research and has become widespread in sports performance training, there is a dearth of scientific enquiries evaluating its effectiveness. The principle of individualization is an essential consideration when designing a resistance training program to optimize the adaptations to training (Borresen and Lambert, 2009; Kiely, 2012). A few studies have shown that training adaptations are enhanced when training is tailored to an individual (Jones et al., 2016). Despite a paucity of interventional studies on autoregulation, several studies have utilized autoregulation training *via* the progressive resistance exercise (PRE) model. The PRE model originated in rehabilitation research and is reported to involve progressively heavier sets of 10 repetitions: one set at 50% of 10RM, one set at 75% 10RM, and one set at 100% 10RM (Delorme et al., 1950).

A daily adjustable progressive resistance exercise (DAPRE) program was implemented with an autoregulated set, which accounted for daily responses in the rehabilitation of a patient (Knight, 1979). This approach allowed for an interactive protocol, which objectively determined the optimal time to increase resistance and the optimal amount of weight during exercise. This provided a more efficient rehabilitation accounting for the individualized reacquisition of strength (Knight, 1990). A specific autoregulatory program derived from the DAPRE method expanded in this concept to meet different training goals of hypertrophy and strength/power and allowed for continual body adaptation through a specific adaptation to imposed demand (SAID) principle (Siff, 2000; Cunanan et al., 2018). This method, termed as autoregulatory progressive resistance exercise (APRE), enhances the previous DAPRE method by introducing training cycles aimed at improving hypertrophy, strength, and power regimes of conditioning. This allows for a continual neuromuscular adaptation by systematically changing the program variables, thus promoting efficient performance gains (Horschig et al., 2014). The autoregulation approach helps to facilitate rapid strength gains without the risk of excessively overloading the joints and tissues, potentially causing injury (Wilson, 2008; Brumitt and Cuddeford, 2015). Without monitoring and adaptation, even the most well-considered protocol potentially becomes irrelevant. Furthermore, physiotherapists face the challenge of dealing with the complexities and heterogenous nature of an individual's recovery and healing process. Meanwhile, adherence and training consistency can enhance adaptation, planned training variation is also essential to ensure relevance on any given day. Therefore, modifiable programming based on relevant feedback is important, which can be achieved using autoregulation (Lorenz and Morrison, 2015).

Mann et al. (2010) adopted an APRE model of periodization and applied it with collegiate athletes. There was a set number of repetitions performed at the percentage of 10RM, 6RM, and 3RM, with athletes self-adjusting the weight after the third set. Athletes performed a maximum number of repetitions to failure during the third set with 100% of their 6RM, determining the load for the fourth set using the number of repetitions completed. This autoregulation method was not only daily adjusted but also decided the weekly changes in load; however, volume and intensity were not controlled due to the inclusion of sets with as many repetitions as possible. These individual differences in volume and intensity could be a reason for the effectiveness of autoregulation training (Siff, 2000). This autoregulation method proved to be effective in increasing maximal bench press and squat strength over 6 weeks in athletes compared to traditional linear periodization (Mann et al., 2010). This autoregulation approach was a modification of the DAPRE system that allows for a more flexible application than more traditional approaches (Rhea et al., 2006). Tables 2, 3 highlight the APRE training routines and the amount of load adjustment following the third set of repetitions to failure.

**Table 2:** Adjusted progressive resistance exercise training routines.

**Set**	**3RM routine**	**6RM routine**	**10RM routine**
0	Warm-up	Warm-up	Warm-up
1	6 repetitions (50% of 3RM)	10 repetitions (50% of 6RM)	12 repetitions (50% of 10RM)
2	3 repetitions (75% of 3RM)	6 repetitions (75% of 6RM)	10 repetitions (75% of 10RM)
3	Repetitions to failure (3RM)	Repetitions to failure (6RM)	Repetitions to failure (10RM)
4	Adjusted repetitions to failure^*^	Adjusted repetitions to failure^*^	Adjusted repetitions to failure^*^

* *Denotes training load must be adjusted according to this table*.

*RM, repetition maximum*.

**Table 3:** Example adjustments for APRE protocols.

**Adjustments for 3RM**		**Adjustments for 6RM**		**Adjustments for 10RM**	
**Repetitions**	**Set 4**	**Repetitions**	**Set 4**	**Repetitions**	**Set 4**
1–2	Decrease 5–10 pounds	0–2	Decrease 5–10 pounds	4–6	Decrease 5–10 pounds
3–4	Same	3–4	Decrease 0–5 pounds	7–8	Decrease 0–5 pounds
5–6	Increase 5–10 pounds	5–7	Same	9–11	Same
7+	Increase 10–15 pounds	8–12	Increase 5–10 pounds	12–16	Increase 5–10 pounds
		13+	Increase 10–15 pounds	17+	Increase 10–15 pounds

*RM, repetition maximum*.

Another study found that autoregulation training significantly increased leg press strength compared to non-linear periodization in novice weightlifters for 12 weeks (McNamara and Stearne, 2010). The autoregulation group was able to choose among the three training sessions of various intensities depending on their energy and motivation levels (10RM, 15RM, or 20RM, repetitions of free weight exercises). Unlike the previous study (Mann et al., 2010), volume and intensity were matched between groups, therefore the autoregulation group had a limited number of exercise selection. Although the autoregulation group performed better in leg press strength, both groups significantly improved, which have been attributed to all participants being new to strength training. Therefore, a rapid improvement in strength due to improved neuromuscular efficiency and motor unit recruitment would be expected (Folland and Williams, 2007). However, improvements in chest press strength were not significantly different between the two groups.

In a physiotherapy case report by Ardali (2014), a DAPRE protocol was implemented alongside standard rehabilitation to maximize the quadriceps muscle strength in a 61-year-old female following the total knee replacement. In phase 1 of the intervention, the emphasis was on edema management, improving the left knee range of motion, and reducing pain over the three sessions. The second phase consisted of two components: (1) a DAPRE protocol aimed at increasing quadriceps strength and (2) functional training aimed at normalizing the altered gait patterns, transfers, and dynamic balance. Patients significantly improved their functional performance and quadriceps strength in the 7 weeks following knee surgery and were able to return to daily living activities without knee pain. The early postsurgical implementation of a DAPRE protocol had no adverse effects and was successful in enhancing the functional performance and muscle strength in this case (Ardali, 2014).

In a physiotherapy case report by Horschig et al. (2014), an APRE model of periodization was investigated using the back squat during the rehabilitation of a 17-year-old male athlete recovering from ACL reconstruction. A secondary aim was to examine the applicability of this method in the transitional period from rehabilitation to S&C for which a current disconnect exists. Starting at 20 weeks postoperatively, the athlete was able to show a 10 pounds daily average increase with the 10RM protocol, a 6 pounds daily average increase during the 6RM protocol, and a 6.3 pounds average increase with the 3RM protocol. A 2RM of 390 lbs was performed in the back squat in the conclusion of the program at 39 weeks postoperatively. The results strengthen the current limited knowledge regarding periodization during the later phases of rehabilitation and the transition back to sport participation time period while at the same time providing new insights for future protocol considerations in rehabilitating athletes. The APRE method of periodization provides an individualized progressive resistive protocol that can be used to safely and effectively increase the strength in both healthy populations and individuals recovering from injury during short-term training cycles (Horschig et al., 2014). Although autoregulation research originated in rehabilitation and these two case reports provide encouraging results, there is a clear lack of longitudinal and interventional research of its effectiveness in physiotherapy.

Autoregulation training should not be considered as a stand-alone periodization model, and it can be applied individually during the periodization in three ways: to adjust load during training, to progress weekly load, or to select daily load prior to training (Klemp et al., 2016; Zourdos et al., 2016a). The modification of training volume with autoregulation has typically been the training variable successfully adopted in studies; however, other training variables have also been examined. The effectiveness of autoregulation of rest intervals in trained men compared to fixed rest intervals has been successfully demonstrated, resulting in greater performance outcomes (Goessler and Polito, 2013). A recent systematic review also concluded that autoregulated rest periods were as good as/better than the structured ones for strength and muscle gain (Henselmans and Schoenfeld, 2014).

## Autoregulation Progression Methods

### Rating of Perceived Exertion

The rating of perceived exertion (RPE) scale has been found to be a reliable measure of not only training session intensity but also specific exercise intensity during a session (Lins-Filho Ode et al., 2012; Kraft et al., 2014). Although most studies have found that RPE is an accurate measure of fatigue during strength training, some have found it is not (Day et al., 2004; Duncan et al., 2006; Gearhart et al., 2009; Tiggemann et al., 2010). However, the more experienced in training someone is, the more accurate an RPE scale becomes, with it being less accurate for younger or novice athletes (McGuigan et al., 2008; Testa et al., 2012). In physiotherapy, an RPE scale offers advantages as it allows intensity monitoring without establishing 1RM, which is contraindicated due to injury (Lorenz and Morrison, 2015). However, the Oddvar Holten curve and other models that exist estimate 1RM without lifting a true 1RM, which are used to establish an estimated 1RM based on submaximal loads taken to failure (Lorenz et al., 2010; Haff and Triplett, 2016). Recommendations have been published for using the Borg RPE scale with resistance band strength training in physiotherapy (Day et al., 2004; American College of Sports Medicine, 2009). A recent study on patients with patellofemoral pain adopted this model with an RPE >6 considered to be desirable (Drew et al., 2017). Although this model may be useful in general population patients only using resistance bands, exercise rehabilitation programs in athletes with tendinopathy typically require the use of much heavier external loads to derive the required adaptations. This suggests that more specific progression models for heavier resistance training in rehabilitation are necessary to achieve optimal progression.

### Repetitions in Reserve

The Reactive Training Manual by Tuchscherer (2008) details an autoregulation training method common in powerlifting based on an RPE scale. The scale allows an individual to evaluate his/her performance based on the number of repetitions they perceive that they could have completed prior to muscular failure. The scale goes from 1 to 10, with 10 being the maximum number of repetitions. This model is like a repetitions in reserve (RIR) model and is considered by some authors to be more appropriate for resistance training than the common RPE methods used in endurance training (Zourdos et al., 2016b). An RPE of 8 indicated that two extra repetitions could be completed, for example, guiding the adjustments in training load. This type of RIR-based RPE scale may have more specificity than the traditional Borg scale RPE, which has shown submaximal scores even when individuals perform exercises to muscular failure (Shimano et al., 2006; Pritchett et al., 2009; Hackett et al., 2012). Therefore, in S&C, it has been suggested this specific RPE is more accurate and superior for assessing the intensity compared to traditional RPE during resistance training (Helms et al., 2016; Hackett et al., 2017; Mansfield et al., 2020).

Recently, a resistance training-specific RPE scale has been demonstrated to be effective for measuring the effort during a training session and subsequently adjusting load (Zourdos et al., 2016b). Like the previous model (Tuchscherer, 2008), it measures RIR, where the individual records an RPE during or after completing a set, with 10 being the maximum, 10RPE = 0RIR. The model allows an individual to estimate the number of additional repetitions remaining at a given load. Consequently, if an achieved RPE is too low or high, training load can be altered accordingly and objectively (Zourdos et al., 2016b). For example, an RPE of 9 or 10 could require a load reduction of 2.5–5 kg. This method is preferable for the load assignment to APRE models as they involve training to failure at predetermined loads, meaning a lack of flexibility in training loads and exertion, whereas the RIR RPE model avoids training to failure (Zourdos et al., 2016b). For example, in APRE studies, athletes perform a maximum number of repetitions to failure during a third set with 100% of their 6RM, with the number of repetitions completed determining the load for the fourth set (Mann et al., 2010). Although this resistance training-specific scale has been validated in experienced and novice weightlifters, its application in rehabilitation such as musculoskeletal physiotherapy has not been evaluated (Zourdos et al., 2016b). Nevertheless, this training-specific RIR-based RPE scale is a potential method to regulate training load in clinical patients prescribed resistance training, such as those undertaking traditional resistance loading programs in tendinopathy (Fairman et al., 2017). Recently, the specific RIR-based RPE model was compared against a traditional 1RM load assignment in resistance trained men. Although both loading types were effective, the RPE strategy provided a small 1RM strength advantage in most participants (Helms et al., 2018). Another study compared the RIR autoregulation approach with a fixed-loading intervention based on 1RM for squat strength in trained men over 12 weeks. Although both groups improved squat performance, the RIR autoregulation group showed significantly greater improvements and was trained at an overall higher intensity (Graham and Cleather, 2019). The results from these studies suggest that RIR as a method of autoregulation training can lead to superior strength gains in athletes compared to traditional fixed approaches. However, as a subjective method, it is less effective compared to the objective methods of autoregulation (Shattock and Tee, 2020).

### Velocity-Based Training

An objective monitoring method during training sessions is velocity-based training (VBT), which has become increasingly popular due to new technology for monitoring repetition velocity (Nevin, 2019). Despite the lack of research on VBT, several models have been developed that determine the intensity based on barbell velocity during lifting and the end of the set based on a predetermined velocity decrease (Gonzalez-Badillo et al., 2011; Dorrell et al., 2019). Technological devices are available to allow quantitative visualization of the effort of an individual, by recording repetition velocity (Jovanovic and Jukic, 2020). Research and practical application of VBT are becoming increasingly popular in S&C and, when VBT is applied systematically, it allows for an immediate feedback, a fatigue control, and the monitoring of biomechanical changes, and can guide the training process (Banyard et al., 2020; Lahti et al., 2020; Martinez-Cava et al., 2020; Perez-Castilla et al., 2020; Sindiani et al., 2020; Suarez-Arrones et al., 2020; Tsoukos et al., 2020). However, for obtaining accurate measurements, individuals must perform fast concentric actions with a good technique, and training adherence and motivation, which may be a barrier for use in rehabilitation (Kawamori et al., 2006). Recently, a VBT intervention was found to increase maximal strength and jump height in the trained men compared with a traditional 1RM percentage-based training approach for 6 weeks (Dorrell et al., 2020). The VBT group achieved these better outcomes and also having a reduction in total training volume compared with the traditional group, indicating a potentially more efficient training method. Despite a dearth of VBT research in physiotherapy, VBT may have a role on musculoskeletal rehabilitation, particularly on athletes who are involved in S&C or transitioning from rehabilitation into S&C (Lorenz and Morrison, 2015).

## Autoregulation in Physiotherapy

### Self-Management in Physiotherapy

Recent UK government policy has emphasized the improvement of outcomes in the National Health Service (NHS) by placing the patient at the center of all decisions taken about them. However, the NHS is also facing financial challenges and the need to deliver gains in efficiency (Littlewood et al., 2013b). Self-management in physiotherapy has been suggested as a solution to these issues by reducing the demand for a regular contact with physiotherapists. Self-management also offers opportunities to individualize care, and there is evidence to suggest that an approach by which patients are encouraged to take responsibility for their own care is no less effective than the treatment requiring regular clinic attendance (Littlewood et al., 2013b). Self-management involves individuals to actively manage their own symptoms, treatments, consequences, and changes to lifestyle, which may be associated with their condition (Barlow et al., 2002). This process is facilitated through a partnership referred to as a therapeutic alliance between the physiotherapist and the patient. The self-management framework consists of components, which are considered as essential for enhancing self-efficacy and facilitating self-management such as skill acquisition, problem solving, self-monitoring, and goal setting (Holman and Lorig, 2004).

### Real-Time Feedback

Evidence suggests that an adherence to the prescribed self-management exercise in physiotherapy patients can be improved by using interventions such as activity monitoring and feedback systems (Peek et al., 2016). Meanwhile, in S&C, feedback methods such as VBT aimed at helping individuals to maintain high exercise velocity in physiotherapy, exercise was often performed too fast in musculoskeletal rehabilitation (Rathleff et al., 2015b). This results in contraction time being too short and too few repetitions, which can impede the recovery due to a low exercise dose (Osteras et al., 2013). As well as the overall load, factors such as adequate contraction time or “time under tension” can influence adaptations such as increasing muscle protein synthesis (Burd et al., 2012). Studies have found that both patients and healthy individuals often do not perform exercises in the predefined contraction time, which affects exercise compliance and outcomes (Faber et al., 2015). This could be particularly important during tendinopathy rehabilitation, where HSRT exercises are performed very slowly to maximize contraction time (Riel et al., 2019a). For example, a common HSRT program requires 8 s (3 s concentric, 2 s isometric, and 3 s eccentric) to complete one repetition (Rathleff et al., 2015a).

A new technology that can provide a real-time feedback on exercises performed with an elastic band is a sensor called BandCizer (BandCizer Aps, Odense, Denmark). It has been validated to quantify contraction time, the number of repetitions performed, and the force used to stretch the band by measuring its thickness (Rathleff et al., 2015b). Data from the sensor can be transmitted to an iPad (Apple Inc., Cupertino, CA, USA), with the BandCizer app providing a real-time exercise feedback (McGirr et al., 2015). It has been found feasible for measuring contraction time and exercise dose during musculoskeletal physiotherapy (Rathleff et al., 2017). Recently, the first RCT investigating BandCizer in musculoskeletal physiotherapy found that the real-time feedback on contraction time resulted in exercises being performed closer to the prescribed dose and induced larger strength gains than the control group (Riel et al., 2016). Although this study was performed in those with patellofemoral pain, the prescribed contraction time was 8 s, the same time as repetitions in the PHP HSRT program. This suggests that providing a similar feedback may be applicable if applied in tendinopathy rehabilitation to improve compliance and adequate loading, which has been identified as a barrier in achieving the desired outcomes (Riel et al., 2019a).

### Pain-Monitoring Models

The common-sense model of self-regulation of health and illness suggests that patients interpret their individual pain response in a way to facilitate the use of exercise training as a management strategy being pivotal (Hale et al., 2007). Persistent beliefs that the pain is associated with tissue damage, and that the rest is required to enable tissue recovery can prevent the successful implementation of rehabilitation (McAndrew et al., 2008). Such an appraisal could lead to activity avoidance behavior, the prevention of necessary rehabilitation engagement, and adherence (Phillips et al., 2012). In this case, reliance is dependent on the adoption of a therapeutic alliance where concerns and worries can be expressed by the patient, with the reassurance offered by the physiotherapist, including an appropriate explanation of the cause of the problem (Littlewood et al., 2012). The appropriate interpretation of physiological symptoms and responses alongside adequate self-monitoring is considered an essential component of self-management (Newman, 2008).

Several studies on tendinopathy have encouraged patients to monitor their pain response during resistance training, *via* the recording of their pain scores in a self-report diary, with an instruction that the pain produced during training is acceptable but should be no worse after training (Jonsson and Alfredson, 2005; Bernhardsson et al., 2011; Littlewood et al., 2016). When the pain response during training decreases, this is regarded as an indication to progress the loaded exercise by increasing the resistance. In contrast to others who have used a numeric pain rating scale, which does not allow the pain to increase >5/10, to guide training progression, the self-monitoring intervention described allows patients to determine what is manageable in terms of symptom response (Holmgren et al., 2012). This decision reflects the individual perceptions of what constitutes acceptability in terms of pain and which differs between the individuals (Littlewood et al., 2012). Littlewood et al. (2016) proposed the SELF study, an RCT, aimed to evaluate the clinical effectiveness of a self-managed single exercise program, which was self-progressed using pain-monitoring vs. usual physiotherapy treatment for rotator cuff tendinopathy. In the 6- and 12-month follow-up, there was no significant difference between the groups. This study suggested that there is no superiority of one intervention over the other in the short-, mid-, or long-term and that a self-management program based around a single self-progressed exercise *via* pain-monitoring appears to be comparable to a usual physiotherapy treatment (Littlewood et al., 2016).

Progression *via* pain-monitoring in Achilles tendinopathy has also been found feasible. Patients with Achilles tendinopathy who are continuing plyometric exercises, such as sprinting, jumping, and hopping, reported pain and function similar to those who stopped all plyometric activities (Silbernagel et al., 2007b). This study identified that plyometric loads are tolerable during tendinopathy rehabilitation in active people, provided they are guided by a pain- and symptom-monitoring model (Silbernagel et al., 2007b). Recently, this model was expanded further in a feasibility cohort study. A pain-guided progressive plyometric hopping intervention was found to be safe and feasible when added to the currently recommended HSRT exercise and education for Achilles tendinopathy in recreational runners (Sancho et al., 2019b). Recreational and elite athletes with tendinopathy will need to adapt their training and rehabilitation parameters such as frequency, intensity, and duration due to persistent pain. This could have significant consequences for athletes as it can restrain them from training for long periods, decreasing fitness levels, and performance (Petraglia et al., 2017). However, there has been a lack of studies on the investigation of solutions to this problem despite the high prevalence of tendinopathy in athletes (Tahririan et al., 2012). The use of pain-monitoring models during rehabilitation may be a possible solution, to ensure that adequate training levels are maintained.

Historically, the rest from pain-provoking activities, namely running and jumping have been recommended as an initial treatment in tendinopathy (Paavola et al., 2000). However, recommendations in the literature are vague when recommending some types of modified rest in which the pain provoking activity should be limited or avoided (Kader et al., 2002; Paavola et al., 2002). As athletes are generally very physically active, an over-long period of rest or a decrease in physical activity due to tendinopathy may have a negative effect on their quality of life and sporting performance (Petraglia et al., 2017). In tendinopathy research, it is now generally accepted that a complete rest period is not helpful, with the loaded exercise required for the healing process and continuing a moderate pain-provoking activity may be beneficial when adjusted appropriately (Silbernagel et al., 2007b, 2011).

### Autoregulation in Tendinopathy

Since the initial research of autoregulation conducted on physiotherapy patients, there has been a lack of further research, particularly longitudinal research, with only case reports appearing in the physiotherapy literature (Ardali, 2014; Horschig et al., 2014). Despite the preponderance of research showing the effectiveness of resistance training for musculoskeletal disorders, it is usually prescribed or investigated using the predetermined protocols (Ciolac and Rodrigues-da-Silva, 2016). Although the importance of identifying methods for enabling patient self-management with the exercise is becoming increasingly emphasized in physiotherapy, the use of autoregulation methods for progressing a self-managed exercise has been given little attention (Peek et al., 2016). A common issue in the use of predetermined exercise programs in physiotherapy is that the prescription and progression of loaded sets and repetitions are often poorly defined and underdosed (Riel et al., 2019a). If the dosage of training loads is not enough, then the mechano-biological stimulus may not be adequate to stimulate healing, resulting in poor outcomes (Khan and Scott, 2009). Previous studies have shown an association between increased exercise dose and the recovery for musculoskeletal disorders (Osteras et al., 2013). An option for increasing training dosage would be to prescribe a larger exercise dose; however, an compliance with exercise can be compromised by a low self-efficacy (McLean et al., 2010). An alternative approach to increase the exercise dosage is to increase the self-efficacy of patients by empowering patients to be in control of their own rehabilitation (Jack et al., 2010). To the authors' knowledge, only two RCTs have investigated autoregulatory resistance training protocols vs. standardized protocols in tendinopathy. A self-dosed protocol in one study on Achilles tendinopathy (Stevens and Tan, 2014) and one study of PHP (Riel et al., 2019a) is compared with the conventional standardized heel-raise protocols according to the work of Alfredson and Rathleff, respectively. Although these studies have not used any true autoregulation methods or distinct load progression, they are still innovative as they allowed the experimental group to self-select the amount of exercise completed as opposed to following a non-flexible protocol.

Riel et al. (2019a) recently investigated this approach in patients with PHP by comparing a 12-week self-dosed HSRT program with a predetermined program. The HSRT program was based on the protocol, which was previously shown more effective than stretching for PHP (Rathleff et al., 2015a). Both groups performed standing heel raises; however, the experimental group was instructed to perform the exercise with the load as heavy as possible but no heavier than 8RM and for as many sets as possible. Participants in the control group were instructed to perform the exercise according to a rigid protocol progressing from 12RM to 8RM. Both groups performed exercises every 2nd day in a 12-week intervention. If participants felt they could perform more repetitions than their load corresponded to (e.g., 10 repetitions when the load was supposed to be 8RM), a backpack with books to add weight was used (Riel et al., 2019a). There was no significant between-group difference in the improvement of Foot Health Status Questionnaire pain after 12 weeks (adjusted MD 26.9 points, 95% CI 215.5–1.7). According to the Global Rating of Change, 24 of 33 in the experimental group and 20 of 32 in the control group were improved (RR = 1.16, 95% CI 0.83–1.64). However, only the four participants achieved the patient's acceptable symptom state, suggesting that more comprehensive interventions are warranted. Although tendinopathy loading programs are typically predetermined, this study indicates that standardized programs are unnecessary if the self-dosed programs aiming to maximize the load are equally effective (Riel et al., 2019a).

Similarly, Stevens and Tan (2014) found that performing a 6-week do-as-tolerated program of the Alfredson eccentric heel-drop exercises led to similar outcomes in Achilles tendinopathy pain and function compared to the standardized 180 repetitions per day protocol. About 28 patients were randomized to either the standard (*n* = 15) or the do-as-tolerated (*n* = 13) intervention for only 6 weeks. The group performing standard training completed 180 repetitions daily, by completing three sets of 15 repetitions, with either the knee fully extended or slightly flexed, two times per day. The experimental group completed the Alfredson protocol, with a guidance that they could choose to complete a repetition volume that was tolerable. All aspects of management were standardized between the groups other than the repetition volume completed. There was a statistically significant within-group improvement in the function for both the groups; however, only the do-as-tolerated group significantly improved the pain at the end of the intervention based on the VISA outcome scores. There were no statistically significant differences between the groups in the completion of the intervention. The mean numbers of repetitions completed per day by the participants were 112 and 166 for the do-as-tolerated and standard groups, respectively (Stevens and Tan, 2014).

Contrary to expectation, the self-dosed HSRT groups in both the studies did not substantially increase the achieved exercise dose compared with a predetermined regimen. Riel et al. (2019a) concluded that these HSRT regimens are not enough to achieve an acceptable symptom state in most people with PHP. Perhaps, this is because heel-raise HSRT alone does not cater for the deficits in a patient's entire strength spectrum, with the addition of other exercise types such as kinetic chain and plyometric exercise being needed to address strength deficits. The use of a “as many sets or repetitions as possible” method did not increase the number of sets performed in the self-dosed groups compared to the predetermined groups. Perhaps, the use of a RIR-based RPE method of autoregulation could be a potential strategy to ensure that an adequate and optimal amount of load is achieved and progressed for each individual patient undertaking HSRT interventions. The use of RPE to dictate the number of repetitions and sets performed as a volume autoregulation method has been found effective to increase the total volume and strength gains in powerlifting athletes but has not been investigated for increasing the strength in tendinopathy rehabilitation (Helms et al., 2018).

## Practical Applications

Although the previously described autoregulated HSRT protocols (Stevens and Tan, 2014; Riel et al., 2019a) were innovative approaches attempting at improving the earlier protocols of Alfredson and Rathleff by allowing an individualized modification, patients did not perform neither an increased number of sets or repetitions nor increased load. Perhaps, the set progression by a RIR approach would be preferable to perform as many sets or repetitions as possible. This could also allow for within-session increases in load, accounting for an individual's daily readiness to train and their own individual factors. The intra-session changes and load achieved due to RIR monitoring could then be used to determine future daily load selection and weekly adjustment. For example, in the self-dosed Riel protocol, the target was to perform 8RM as heavy as possible for as many sets as possible at a specific load. However, if the patient was to record a 10RIR on the first set, then the load could be lowered to ensure the completion of the intended training volume. Intra-session external load adjustments could also be made using the RIR scale based upon the predetermined RIR goals. For example, if a target RIR is set at 5–6 and a RIR of 7 is recorded, a 2.5 kg load decrease could occur, and for a RIR of 9, a 5 kg load decrease occurs. Similarly, a RIR of 3–5 would require an adjustment of 2.5 kg increase in load, and an RIR of 1–2, a 5 kg increase (Table 4).

**Table 4:** Theoretical load adjustment during autoregulated tendinopathy heel-raise resistance training based on the representation of rating of perceived exertion and corresponding repetitions in reserve vales.

**Warm-up if required**			
**Set 1: Based on load lifted during last set of last session**			
**Set 2–4: Load adjusted per set based on RPE/RIR values during previous**			
**set**			
**RPE**	**RIR**	**Description of perceived effort**	**Example load adjustment**
10	0	Maximum effort, cannot increase load or repetitions	−5 kg
9.5	0.5	Cannot complete more repetitions but could increase load	−2.5 kg
9	1	1 repetition remaining	Maintain load
8.5	1.5	1–2 repetitions remaining	Maintain load
8	2	2 repetitions remaining	Maintain load
7.5	2.5	2–3 repetitions remaining	+2.5 kg
7	3	3 repetitions remaining	+2.5 kg
5–6	4–6	4–6 repetitions remaining	+5 kg
3–4	6+	Light effort	+7.5 kg
1–2	10+	Little to no effort	+10 kg

Following an initial set at a load determined by the previous session, sets 2–5 can be adapted in load based on RIR, with the load in the fifth set determining the starting load of the next session, as in the previous APRE protocols. Many tendinopathy resistance training protocols stipulate an increase of load increments of 2.5–5 kg between the sessions if there is no increase in pain. Adopting this intra-session autoregulated approach based on RIR would allow for individualized loading in response to an individual's daily readiness to train and their individual factors. This individualized approach to load adjustment may allow for a more specific load progression than the standardized protocols, which do not adjust the load during the sessions, potentially improving the outcomes of individuals to the intervention. The rest could also be autoregulated as this has been shown no less effective than the predetermined rest protocols for increasing strength and hypertrophy (Henselmans and Schoenfeld, 2014). A comparison of how this theoretical autoregulated protocol would be compared to the Rathleff and Riel protocols for PHP is presented in Table 5. The example provided is specific to HSRT for PHP, with autoregulation methods requiring feasibility and effectiveness investigations across all lower limb tendinopathies.

**Table 5:** Descriptors of Rathleff, Riel and autoregulated interventions.

**Descriptor**	**Exercise program**		
	**Rathleff**	**Riel**	**Autoregulated**
Load magnitude	Weeks 1 + 2: 12RM Weeks 3 + 4: 10RM Weeks 5–12: 8RM	As heavy as possible, no heavier than 8RM	8RM
Monitoring per set	Not reported	Not reported	RPE RIR scale—adjust load based on response
Load progression and volume	Increase weight in backpack—not recorded	Increase weight in backpack—not recorded	Adjust weight per set based on RPE RIR scale—record weight
Number of repetitions per set	Weeks 1 + 2: 12 Weeks 3 + 4: 10 Weeks 5–12: 8	8, depending on load	8
Number of sets	Weeks 1 + 2: 3 Weeks 3 + 4: 4 Weeks 5–12: 5	As many as possible	5
Rest between sets	2 min	2 min	As much as needed
Session frequency	3/week		
Program duration	12 weeks		
Contraction modes per repetition	3 s concentric, 2 s isometric, 3 s eccentric		
Rest between repetitions	Nil		
Time under tension	Weeks 1–2: 8 s/repetition, 96 s/set, 288 s/training session Weeks 3–4: 8 s/repetition, 80 s/set, 320 s/training session Weeks 5–12: 8 s/repetition, 64 s/set, 320 s/training session Total over 12 weeks: 13 216 s	8 s/repetition, 64 s/set, Total over 12 weeks: varies between participants depending on number of sets performed	8 s/repetition, 64 s/set, 320 s/training session total over 12 weeks: 13,440 s
Muscle failure	Yes	Yes	Not mandatory
Range of motion	Full range of motion		
Between session recovery	48 h		
Exercise description	Standing heel raise, with forefoot on a step and a towel placed underneath the toes to dorsiflex them throughout the exercise. With a fully extended knee, the participant performed a heel raise		

Despite the potential efficacy of such an autoregulation approach in tendinopathy, several potential limitations to its practical application exist. Although this theoretically autoregulated protocol only includes a progressive HSRT heel-raise exercise, future interventional studies should also consider adding plyometric exercises and exercises with high kinetic chain activation such as squats and deadlifts, progressed in the same autoregulated manner, to address potential strength spectrum deficits (Boren et al., 2011; Reiman et al., 2012). Accurate use of RPE scales has been shown to be higher in those with more training experience, and practice is required to become competent in using this method (McNamara and Stearne, 2010). The application may, therefore, be more suitable for athletes with tendinopathy who have significant training experience. The use of the previous sessions loading parameters to dictate the starting load for the following sessions does not consider daily readiness, which needs to be monitored at an individual level by each patient. Due to higher fatigue levels on any given day, the starting load may need to be subjectively reduced, which again will require an experience to become proficient in making such judgments. Guidance and intervention instruction by a physiotherapist with training in the clinical application of resistance training principles and methods could improve the efficacy and safety efficacy of applying the intervention methods, especially with participants who lack training experience. The use of an RPE RIR progression method will naturally have an element of unreliability due to its subjectivity, and objective autoregulation methods such as VBT have been found slightly more effective when compared against each other (Shattock and Tee, 2020). However, its advantages include an easier implementation while also being a potential method for increasing patient self-efficacy and allowing the self-management in the home setting without the need for expensive equipment, making it a more practical and cost-effective choice.

## Conclusion

This review has provided an overview of individual factors in tendinopathy, the current resistance training protocols in lower limb tendinopathy and their limitations, and autoregulation methods culminating in a proposed example of the implementation of autoregulation under the current tendinopathy HSRT protocols. While current resistance training protocols are considered as the gold standard treatment intervention for a range of tendinopathies, they are not without limitations as they fail to address an individual's entire strength spectrum and the principle of individualization, resulting in inadequate long-term outcomes. This review provides an example of how these factors may be addressed through autoregulation methods, which may improve the outcomes for tendinopathy, which is a leading global cause of musculoskeletal pain and disability. Previous literature studies have demonstrated how the adoption of autoregulation methods in resistance training can improve strength gains and sports performance in healthy athletes. However, there has been a dearth of research applying these autoregulation principles to musculoskeletal rehabilitation despite the methodology originating in physiotherapy research. This review demonstrates how applying these autoregulation methods to widely adopted resistance training protocols in tendinopathy allows for tailoring the exercise dosage and considering the myriad of potential patient individual factors, which are not accounted for in the standardized and predetermined protocols. However, as a narrative review based on the authors' opinion with no interventions investigated, this review has limitations in terms of clinical applicability. Therefore, further interventional research is required to investigate the safety, feasibility, and efficacy of these methods in improving the outcomes of resistance training interventions in tendinopathy rehabilitation.

A key point from this review is noted that autoregulation should be used as a replacement for current interventions, but that it is a context-specific approach and may be used as a modification strategy to the current resistance training protocols in tendinopathy. Eccentric-only and HSRT protocols have been successfully implemented in lower limb tendinopathy rehabilitation around the world for two decades. However, despite their moderate success, a modification through improved prescription, monitoring, and progression based on the principle of individualization may lead to more optimal, individually tailored, and efficacious interventions. For several years, leading tendinopathy clinicians and researchers have been advocating that tendinopathy rehabilitation must be individualized to improve long-term outcomes. However, this has not been addressed, with predetermined and standardized protocols remaining the mainstay of tendinopathy rehabilitation, both clinically and in interventional research methods. It is clear from the recent literature studies that a paradigm shift in the resistance training application in lower limb tendinopathy is required, attempting to improve clinical outcomes for patients, which remain inadequate in the long term. Perhaps, a slight adaptation of these long-standing resistance training interventions through autoregulation methods may pave the way forward for ascertaining the most optimal and efficacious approach for tendinopathy rehabilitation, which has yet to be elucidated.

## Author Contributions

IB conceptualized the work, wrote the first draft of the manuscript, revised the manuscript, and approved the final manuscript.

## Conflict of Interest

The author declares that the research was conducted in the absence of any commercial or financial relationships that could be construed as a potential conflict of interest.

## Publisher's Note

All claims expressed in this article are solely those of the authors and do not necessarily represent those of their affiliated organizations, or those of the publisher, the editors and the reviewers. Any product that may be evaluated in this article, or claim that may be made by its manufacturer, is not guaranteed or endorsed by the publisher.
